# Retrointegration2023—Papers from the 7th International Conference on Retroviral Integration

**DOI:** 10.3390/v17070879

**Published:** 2025-06-23

**Authors:** Alan N. Engelman, Duane P. Grandgenett, Goedele N. Maertens, Kristine E. Yoder, Mamuka Kvaratskhelia

**Affiliations:** 1 Department of Cancer Immunology and Virology, Dana-Farber Cancer Institute, Boston, MA 02215, USA; 2 Department of Molecular Microbiology and Immunology, School of Medicine, Saint Louis University, St. Louis, MO 61304, USA; duane.grandgenett@health.slu.edu; 3 Department of Infectious Disease, Imperial College London, London SW7 2AZ, UK; g.maertens@imperial.ac.uk; 4 Department of Cancer Biology and Genetics, The Ohio State University College of Medicine, Columbus, OH 43210, USA; kristine.yoder@osumc.edu; 5 Division of Infectious Disease, Anschutz Medical Campus, University of Colorado, Aurora, CO 80045, USA; mamuka.kvaratskhelia@cuanschutz.edu

The integration of retroviral DNA into host chromosomal DNA is a landmark event that demarcates the transition from the early steps of virus replication to post-integration gene expression and the assembly of new virus particles. Becoming a permanent part of the host cell’s genome, transcriptionally latent proviruses are impervious to highly active antiretroviral therapy and present the greatest barrier to a cure for HIV. Integrase, the viral enzyme that mediates integration, has risen in prominence to a high-value antiretroviral target over the past two decades, with strand transfer inhibitors now widely prescribed for people living with HIV. Allosteric integrase inhibitors (ALLINIs), which potently inhibit virus particle morphogenesis, are being evaluated in phase II clinical trials and are also investigated for “block-and-lock” cure strategies. Papers addressing these subjects and other integration-related research topics were presented and discussed at the 7th International Conference on Retroviral Integration, which was held in Boulder, Colorado during the summer of 2023. In this Editors’ overview, we discuss the papers since published in this dedicated *Viruses* Special Issue, and briefly touch upon other talks of significant interest.

Retrointegration2023 marked the seventh time that the retroviral community had convened at an international conference dedicated to the science of integrase and viral DNA integration. We five served as the meeting co-organizers, with Dr. Kvaratskhelia serving as the local lead organizer. The conference consisted of seven oral sessions and one poster session, with 36 talks given by invited speakers and 11 short talks selected from submitted abstracts (Table 1). Four papers were published as part of the *Viruses* Special Issue [1,2,3,4]. One of these manuscripts, which expanded upon Dr. Grandgenett’s outro presentation at the end of the conference (Table 1), gave an overview of the field of retroviral integration as well as details of the preceding six meetings [2]. We recommend this paper to readers interested in the history of the field or these dedicated meetings, as well as in the significant field advances that were accomplished leading up to the 2023 conference.

The paper by Sun and Kessl gave an overview of the development and optimization of quinoline-based ALLINIs [1,5]. Also known as LEDGINs (for LEDGF–integrase site inhibitors) [6], NCINIs (for non-catalytic integrase inhibitors) [7], and INLAIs (for IN-LEDGF allosteric inhibitors) [8], these compounds bind the integrase catalytic core domain (CCD) dimer interface at the same location as the cellular integration cofactor lens epithelium-derived growth factor (LEDGF)/p75 [6,9]. CCD-engaged ALLINIs in turn bind the C-terminal domain (CTD) of a separate integrase multimer, inducing the formation of linear and branched-chain integrase–ALLINI–integrase copolymers [10,11,12]. Integrase binds genomic RNA in virions, which is critical for incorporating viral ribonucleoproteins into mature HIV-1 capsid cores [13]. The disruption of integrase–RNA binding, by treating virus-producer cells with ALLINIs or via mutagenesis of specific integrase amino acid residues, produces an eccentric viral phenotype with ribonucleoproteins situated outside the confines of the capsid shell [7,13,14]. Such virions are defective for reverse transcription in the next round of infection, accounting for the potency of ALLINIs in inhibiting the late stage of HIV-1 replication [7,8,14,15].

ALLINIs were the subject of several other talks at the meeting. The propensity of ALLINIs to aggregate/polymerize integrase had, to date, limited the resolution of ALLINI–integrase X-ray cocrystal structures involving proteins that minimally harbored the CCD and CTD. Peter Chereapnov circumvented this bottleneck by reversing the order of the CTD and CCD in the recombinant integrase protein, which intramolecularly restricted ALLINI–integrase interactions [16], while Kushol Gupta utilized separate CCD and CTD protein fragments to obtain well-diffracting crystals [17]. These approaches have since significantly informed the structural basis of resistance to ALLINIs, including the clinical candidate pirmitegravir [18,19]. In his talk, Kyungjin Peter Kim described the development and pre-clinical testing of pirmitegravir [20].

HIV-1 integration favors genes and gene-dense regions of chromosomes [21], and integration in ALLINI-treated cells shifts towards gene-poor regions [22,23]. Such proviruses, formed in the presence of ALLINIs, are comparably less activatable by latency reversing agents [22], indicating that ALLINIs could be part of a block-and-lock cure strategy [24]. In his talk, Zeger Debyser described the potential utility of ALLINIs as block-and-lock agents [25,26]. The clearest vision for such compounds is to irreversibly silence already-integrated proviruses [27]. Because ALLINIs need to be present prior to integration to exert their retargeting and associated silencing effects, it is currently unclear how ALLINIs might best be formulated with other block-and-lock compounds to treat people living with HIV.

Guedán et al. described the relocalization of nuclear CPSF6 in response to HIV-1 infection in cell lines, as well as in primary macrophages [3]. CPSF6 is a member of the heterotetrameric CFIm complex which, together with its CPSF5 partner, regulates the positions of polyadenylation in mRNA [28]. CPSF6 harbors a Phe-Gly motif within a prion-like domain that directly binds capsid [29,30], and CPSF6 has been shown to influence the nuclear import of HIV-1 [31,32] and post-entry trafficking for integration targeting [31,33,34]. Prior work has shown that HIV-1 infection/capsid binding also reorganizes CPSF6 from a largely pan-nuclear staining pattern to discrete puncta that colocalize with nuclear speckles [35,36,37,38]. In her talk, Kate Bishop described the comparatively long-lived nature of HIV-1-induced CPSF6 puncta in non-dividing cells such as growth-arrested HeLa cells and primary macrophages [3]. Interestingly, CPSF6 was observed to partially colocalize with nuclear speckles independent of HIV-1 infection in primary CD4+ T cells [3], which has been observed in other cell types such as NCM460 cells [39]. CPSF6 possesses liquid–liquid phase separation activity [39,40,41], which HIV-1 has seemingly usurped to complete the early steps of virus replication and to help evade detection by cellular innate immune sensors [42,43].

The first cellular protein shown to bind HIV-1 integrase, SMARCB1, was named INI1 at the time for “integrase-interactor 1” [44]. The role of INI1 in HIV-1 replication has been challenging to pinpoint, likely due to the general requirement of SMARCB1 expression for cell viability [45] and the pleiotropic nature of the replication defects associated with INI1 binding-defective HIV-1 integrase mutant viruses [46]. Recent findings from the Kalpana laboratory have determined that the part of INI1 that binds integrase, called Rpt1 for Repeat 1, structurally mimics TAR RNA, which forms a stem–loop structure in the 5′ untranslated region of HIV-1 RNA for transcriptional activation by the viral Tat protein [4,47]. In this way, virion INI1 may act as a placeholder for downstream RNA interactions with integrase to ensure proper viral ribonucleoprotein complex incorporation into HIV-1 capsids. Plausibly, small molecule modulators of integrase–INI1 interactions may disrupt integrase–RNA interactions and virion morphogenesis, akin to the results observed for ALLINIs [4].

Although not associated with the papers in this Special Issue, here we will highlight a few other talks, due to the impact of their findings. In Session 1, Dmitry Lyumkis described the cryogenic-electron microscopy (cryo-EM) structure of the HIV-1 integrase tetramer in the absence of nucleic acid, which included an exposed ionic interface that may very well mediate RNA binding [48]. The structure also suggested a plausible model for how integrase tetramers may morph into higher order multimers engaged with viral DNA for integration [49]. In Session 3, Hans-Georg Kräusslich described the use of advanced cryo-electron tomography techniques to visualize the progression of HIV-1 infection in primary human macrophages. HIV-1 cores could be visualized in the cytoplasm, proximal to nuclear pore complexes, and imported into the cell nucleus. The work showed that HIV-1 nuclear import oftentimes deforms or “cracks” the nuclear pore complex, apparently to accommodate the comparatively large size of the viral core [50]. In Session 4, Stephan Yant described the development of lenacapavir, the first-in-class long-acting capsid inhibitor that has since been shown to effectively prevent HIV-1 transmission [51,52]. In Session 7, Frederic Bushman gave a succinct overview of the effects of integrating retroviral vectors for human T cell therapy. While a small percentage (2.3%) of the 783 individuals analyzed developed secondary malignancies, none of these were directly linked to the position of vector integration, highlighting the general safety of the approaches [53]. Finally, in Session 8, Mary Kearney described the differences in the makeup of proviral reservoirs in adult versus adolescent individuals, which could have ramifications for eventual HIV cure strategies based on patient age [54].

## Figures and Tables

**Table 1:** Talks presented at the Retrointegration2023 meeting.

Session	Speaker	Title
Keynote Talk	Peter Cherepanov	Retroviral DNA integration through the lens of structural biology
1—Retroviral Integrases: Structure and Function	Min Li	HIV intasomes: Where we are and where we are going
	Dmitry Lyumkis	Implications for integrase functional plasticity from the structure of the HIV-1 integrase tetramer
	Krishan Pandey	Molecular determinants of the Rous sarcoma virus intasome assembly
	Kushol Gupta	Structural consequences of resistance mutations on the formation of ALLINI-induced branched polymers of HIV-1 integrase
	Kristine Yoder	DNA strand breaks and gaps target PFV intasome binding and catalysis
	Chandravanu Dash	An optimal substrate for HIV-1 preintegration complex mediated viral DNA integration
	Stephen Goff	Working overtime: Jump-starting provirus transcription, redirecting sites of integration, and activating DNA damage repair pathways
	Wesley Sundquist	Reconstitution and characterization of a cell-free system for HIV-1 capsid-dependent replication and integration
2—Integrase–Host Factor Interactions	Monica Roth	Studies of the common binding motif BRD3 ET domain: Polymorphic structural interfaces with host/viral proteins and small molecules
	Goedele Maertens	Investigating the role of PP2A-B56 in establishing HTLV-1 infection
	Ganjam Kalpana ^1^	INI1/SMARCB1 IN binding domain mimicry to TAR RNA and its influence on viral late events and particle morphogenesis: Development of novel class of INI1-derived inhibitors
	Marina Lusic	HIV-1 integration into R-loop enriched genomic regions is mediated by Aquarius helicase of the Intron Binding Complex
	Henry Levin	The role of LEDGF in transcription is intertwined with its function in HIV-1 integration
(Selected short talks)	Joshua Hope	The rules of engagement between lentiviral integration machinery and chromatin
	Ross Larue	Single molecule visualization of intasome assembly
	Arpa Hudait	Multiscale simulations of HIV-1 capsid nuclear entry and host factor interactions
3—Nuclear Import of HIV-1 Cores/Preintegration Complexes	Hans-Georg Kräusslich	Capsid as key orchestrator of early HIV-1 replication
	Vinay Pathak	Mechanisms of HIV-1 core uncoating, nuclear import kinetics, and integration site selection
	Kate Bishop ^1^	HIV-1 requires capsid remodelling at the nuclear pore for nuclear entry and CPSF6 binding
	Edward Campbell	Distinct utilization of nuclear import pathways allows HIV-1 integration into transcriptionally active regions of the chromatin
	Ashwanth Francis	Live-cell imaging of HIV-1 nuclear transport and association with nuclear speckles
(Selected short talks)	João Mamede	Fluorescent labeled CA correlates progressive uncoating from the cytoplasm to the nucleus to productive HIV infection in primary cells
	Melissa Kane	Effects of the cyclophilin homology domain of RanBP2 on HIV-1 infection and Mx2 activity
4—HIV-1 Integrase Inhibitors and Novel Antiretroviral Compounds	Kyungjin Peter Kim	The Fellowship of the Ring: Quest to develop Pirmitegravir, a novel potent and safe HIV-1 allosteric integrase inhibitor (ALLINI)
	Jacques Kessl ^1^	Optimizing the binding of substituted quinoline ALLINIs within the HIV-1 integrase oligomer
	Stephen Yant	Lenacapavir: A first-in-class, long-acting HIV capsid inhibitor for treatment and prevention
	Daniel Adu-Ampratwum	Developing novel small molecules as inhibitors targeting HIV-1 integrase and capsid proteins
	Eric Gillis	Potent long-acting inhibitors targeting HIV-1 capsid based on a versatile quinazolin-4-one scaffold
	Mark Underwood	Second generation integrase inhibitor resistance in the clinic: Dolutegravir resistance mechanisms and structural underpinnings
(Selected short talks)	Yuta Hikichi	Mutations outside integrase lead to high-level resistance to integrase strand transfer inhibitors
	Jose Dekker	HIV-1 3′-polypurine tract mutations confer dolutegravir resistance by switching to an integration-independent replication mechanism via 1-LTR circles
	Roberto DiSanto	New small molecule derivatives as dual inhibitors of the HIV-1 integrase catalytic site and integrase-RNA interactions
	Szu-Wei Huang	Sub-stoichiometric drug to HIV-1 capsid ratio enables ultra-potent antiviral activity of lenacapavir
5—Poster Session	Not applicable	Not applicable
6—Retrotransposons and Serine Integrases	Suzanne Sandmeyer	Ty3: We should have known it wouldn’t be random
	David Garfinkel	Ty1 Gag stories: mechanism of copy number control, domestication of a restriction factor, and an interchangeable prion-like domain
	Phoebe Rice	Large serine integrases: how do they know which way to go?
(Selected short talks)	Eric Arts	Evidence of significantly reduced HIV proviral integrants within genes and increased integration into transcriptionally silent elements in HIV-1 infected individuals failing an INSTI treatment regimen with or without INSTI resistance mutations
	Ariberto Fassati	Functional mapping of integration sites connected to latent HIV-1 infection
7—Retroviral Integration Site Selectivity	Frederic Bushman	Retroviral DNA integration: Target site selection and genomic consequences
	Charles Bangham	HTLV-1 integration site: Impact on viral persistence and host chromatin structure and expression
	Alan Engelman	CPSF6 liquid-liquid phase separation determines higher-order capsid binding, nuclear core incursion, and HIV integration targeting
	Zeger Debyser	The chromatin landscape of the HIV provirus determines its transcriptional state. Implications for a functional block-and lock cure strategy
8—Latency	Mary Kearney	Divergent populations of infected naïve and memory CD4+ T cell clones in children on ART
	Mathias Lichterfeld	Chromosomal integration sites as biomarkers of HIV-1 reservoir cell selection
	Frank Maldarelli	Anatomic distribution of HIV-infected cells after long term antiretroviral therapy
	Duane Grandgenett ^1^	Concluding remarks: Retrovirus integrase, integration, HIV-1 integrase inhibitors

^1^ These authors’ published work related to their presentations as part of this Special Issue.
